# Multi-Donor Longitudinal Antibody Repertoire Sequencing Reveals the Existence of Public Antibody Clonotypes in HIV-1 Infection

**DOI:** 10.1016/j.chom.2018.05.001

**Published:** 2018-06-13

**Authors:** Ian Setliff, Wyatt J. McDonnell, Nagarajan Raju, Robin G. Bombardi, Amyn A. Murji, Cathrine Scheepers, Rutendo Ziki, Charissa Mynhardt, Bryan E. Shepherd, Alusha A. Mamchak, Nigel Garrett, Salim Abdool Karim, Simon A. Mallal, James E. Crowe, Lynn Morris, Ivelin S. Georgiev

**Affiliations:** 1 Program in Chemical & Physical Biology, Vanderbilt University Medical Center, Nashville, TN, USA; 2 Vanderbilt Vaccine Center, Vanderbilt University Medical Center, Nashville, TN, USA; 3 Department of Pathology, Microbiology, and Immunology, Vanderbilt University Medical Center, Nashville, TN, USA; 4 Center for Translational Immunology and Infectious Diseases, Vanderbilt University Medical Center, Nashville, TN, USA; 5 Center for HIV and STIs, National Institute for Communicable Diseases, Johannesburg, South Africa; 6 Faculty of Health Sciences, University of the Witwatersrand, Johannesburg, South Africa; 7 Department of Biostatistics, Vanderbilt University School of Medicine, Nashville, TN, USA; 8 ATRECA, Inc, 500 Saginaw Dr., Redwood City, CA, USA; 9 Centre for the AIDS Programme of Research in South Africa (CAPRISA), University of KwaZulu-Natal, Durban, South Africa; 10 Department of Epidemiology, Mailman School of Public Health, Columbia University, New York, NY, USA; 11 Division of Infectious Diseases, Vanderbilt University Medical Center, Nashville, TN, USA; 12 Institute for Immunology and Infectious Diseases, Murdoch University, Murdoch, WA, Australia; 13 Department of Pediatrics, Vanderbilt University Medical Center, Nashville, TN, USA; 14 Department of Electrical Engineering and Computer Science, Vanderbilt University, Nashville, TN, USA

**Keywords:** HIV-1, antibodies, antibody repertoire, next-generation sequencing, B cells, public antibodies, systems immunology, immunology, computational biology

## Abstract

Characterization of single antibody lineages within infected individuals has provided insights into the development of Env-specific antibodies. However, a systems-level understanding of the humoral response against HIV-1 is limited. Here, we interrogated the antibody repertoires of multiple HIV-infected donors from an infection-naive state through acute and chronic infection using next-generation sequencing. This analysis revealed the existence of “public” antibody clonotypes that were shared among multiple HIV-infected individuals. The HIV-1 reactivity for representative antibodies from an identified public clonotype shared by three donors was confirmed. Furthermore, a meta-analysis of publicly available antibody repertoire sequencing datasets revealed antibodies with high sequence identity to known HIV-reactive antibodies, even in repertoires that were reported to be HIV naive. The discovery of public antibody clonotypes in HIV-infected individuals represents an avenue of significant potential for better understanding antibody responses to HIV-1 infection, as well as for clonotype-specific vaccine development.

## Highlights

• Within-donor longitudinal antibody repertoire to HIV-1 infection was analyzed by NGS
• Public antibody clonotypes shared among multiple HIV-infected individuals were uncovered
• A public antibody clonotype shared by three donors was confirmed to be HIV reactive
• Antibody sequences from HIV-naive repertoires are similar to known HIV antibodies

## Graphical Abstract

**Figure:**
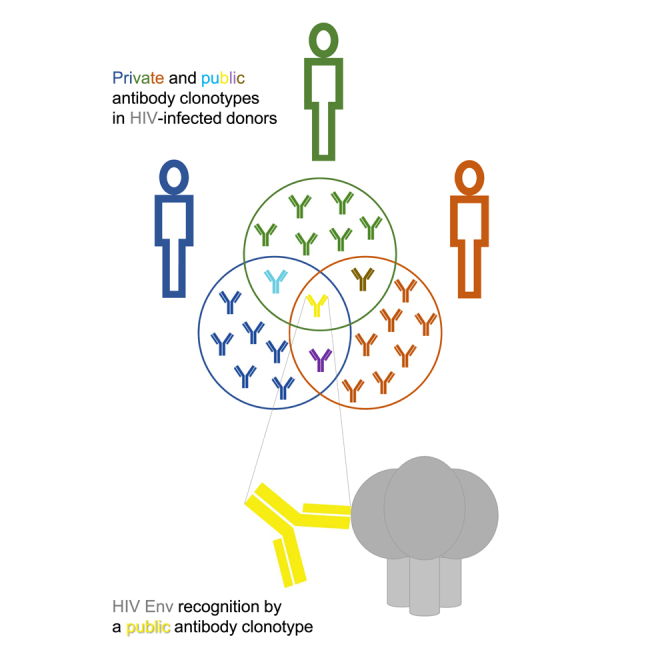

## Teaser

The overall antibody repertoires of HIV-infected subjects are considered to be unique. Setliff et al. analyze the longitudinal antibody repertoire of HIV-1-infected individuals to uncover the existence of “public” HIV-reactive antibodies in multiple subjects. Antibody sequences with high identity to known HIV-reactive antibodies were identified even in HIV-naive repertoires.

## Introduction

The HIV-1 envelope glycoprotein (Env) mediates receptor recognition and viral fusion and serves as the sole target of the neutralizing antibody response (Pancera et al., 2014, Ward and Wilson, 2015). The developmental pathway of Env-specific antibodies has been probed previously using high-throughput sequencing (Bonsignori et al., 2016, Doria-Rose et al., 2014, Huang et al., 2016, Liao et al., 2013, Wu et al., 2011), but such analyses have focused on single broadly neutralizing antibody (bNAb) lineages after infection. However, bNAbs comprise only a fraction of the antibody response within a given individual, which also includes antibodies with limited or no breadth. These diverse antibodies are subject to viral selection pressures and host constraints, target a variety of epitopes on Env, and potentially possess functions other than neutralization (Ackerman et al., 2016, Burton and Mascola, 2015, Corey et al., 2015, Horwitz et al., 2017). More generally, thorough and large-scale profiling of the repertoire-wide antibody response during the course of natural infection remains a predominantly unexplored area of investigation and an unmet need in HIV-1 research. Indeed, the extensive evidence of the global effects that HIV-1 has on the adaptive immune system, including hypergammaglobulinemia (De Milito et al., 2004), CD4+ T cell abnormalities (Kaufmann et al., 2007, Palmer et al., 2004, Zhang et al., 2004), and defective CD8+ T cell function (Harrer et al., 1996, Rinaldo et al., 1995), motivates efforts to understand the dynamics of the antibody repertoires of HIV-infected individuals.

Although putative bNAb precursors have been discovered in HIV-naive repertoires (Jardine et al., 2016, Yacoob et al., 2016), it is unclear how the antibody repertoires of HIV-infected individuals change from the time before infection through different stages of infection. Furthermore, while ontogeny and structural studies of HIV-reactive antibodies have revealed convergence at the structural level in multiple donors (Scheid et al., 2011, Wu et al., 2011, Zhou et al., 2015), the overall differences and similarities in the antibody repertoires of HIV-infected donors have not been characterized. Due to the diversity of potential target epitopes on Env, as well as the potentially infinite antibody sequence space resulting from gene recombination and affinity maturation, it could be expected that the antibody repertoire of each individual might be unique. Yet public antibody clonotypes that are shared among multiple individuals have been observed previously for dengue infection (Parameswaran et al., 2013), after influenza vaccination (Jackson et al., 2014), and in other immune settings (Arentz et al., 2012, Henry Dunand and Wilson, 2015, Pieper et al., 2017, Trück et al., 2015). However, in the context of HIV-1 infection the potential for public antibodies has not been explored.

To better understand antibody repertoire dynamics throughout HIV-1 infection, we performed antibody repertoire sequence analysis to examine characteristics of the pre- and post-infection repertoires of multiple donors. To that end, we longitudinally sequenced the global immunoglobulin heavy chain repertoires of six South African donors from the Centre for the AIDS Programme of Research in South Africa (CAPRISA) from before infection through acute and chronic infection. We also performed paired heavy and light chain sequencing of the Env-specific post-infection repertoires of two additional CAPRISA donors. The resulting analysis provides insights into how antibody repertoires of different individuals are reshaped during the course of HIV-1 infection.

## Results

### CAPRISA Donor Samples

Antibody variable genes in peripheral blood cell samples from three time points, categorized as pre-infection, 6 months post infection (mpi), or 3 years post infection (ypi), were sequenced for each of six CAPRISA donors (Table S1). The pre-infection time points ranged from 30 to 2 weeks before infection, with the exception of donor CAP322, for whom the earliest available sample was at 2 weeks post infection. All CAPRISA donors were infected with clade C viruses (Rademeyer et al., 2016) but exhibited diverse neutralization phenotypes, including substantial variation in neutralization breadth between 0% and 61% on a representative panel of diverse HIV-1 strains (Table S1). For the three donors with demonstrable neutralization breadth (CAP287, CAP312, and CAP322), we also performed neutralization fingerprinting analysis (Doria-Rose et al., 2017, Georgiev et al., 2013) to delineate the epitope specificities of broadly neutralizing antibodies at the 3 ypi time points. These three donors were predicted to possess different types of antibody specificities (Table S2). Taken together, the observed differences in neutralization phenotypes for the six donors indicated diversity in the types of antibody specificities present in each donor.

### High Turnover of Antibody Repertoires During the Course of HIV-1 Infection

**Figure 1:**
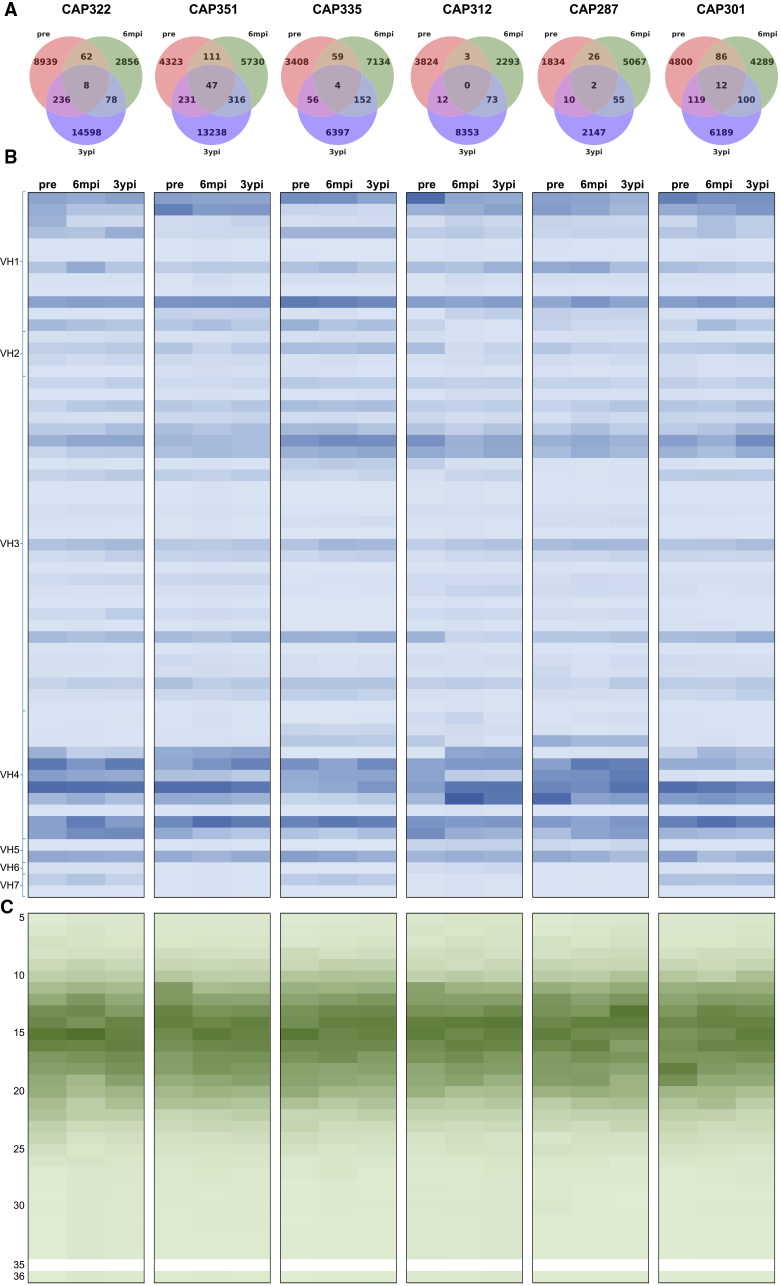
Within-Donor Longitudinal Antibody Repertoire Analysis from Pre-infection through Chronic HIV-1 Infection (A) For each donor, the number of clonotypes unique to each time point is shown, as well as the clonotypes shared between two or all three time points. (B) Heatmap of V-gene usage by donor and time point. For each time point of each donor, the number of clonotypes using each V_H_ gene (excluding orphan genes) was summed and the *Z* score was calculated. *Z* scores range from −0.81 (light blue) to 4.26 (dark blue). (C) Heatmap of CDRH3 amino acid length by donor and time point. For each time point of each donor, the number of clonotypes of each CDRH3 length was summed and the *Z* score was calculated. No sequences had a CDRH3 length of 35 in any sample in this study. *Z* scores range from −0.90 (light green) to 2.54 (dark green).

After sequencing the antibody heavy chain variable gene regions from all three time points for each of the six donors (STAR Methods), we first investigated how repertoire composition changed over time. We began by determining clonal family membership for each observed V(D)J sequence using V-gene assignment, J-gene assignment, junction length, and junction identity (STAR Methods). All time points from each donor were included during clonal family assignment so that closely related sequences from different longitudinal samples could be assigned to the same clonotype. Clonal family assignment revealed the number of clonotypes (groups of sequences resulting from clonal family assignment) in each donor to range from 9,141 to 26,777, with a total of 103,475 unique clonotypes across all six donors (Table S3). Few clonotypes spanned multiple time points within a donor, with the majority of clonotypes belonging to a single time point (Figure 1A). While some clonotypes were present in all three time points within individual donors, this finding was rare, typically representing only ∼0.08%–1.16% of all clonotypes, where clonotypes observed in pre-infection and 3ypi samples, but not 6mpi, were counted toward the number of clonotypes across all three timepoints (Figure 1A). Clonotypes spanning any two adjacent time points were also rare, although somewhat more frequent than those that spanned all three time points, representing ∼0.52%–1.78% of all clonotypes (Figure 1A). Interestingly, although sequence turnover was high, antibody heavy chain variable gene usage distributions remained predominantly constant over the course of infection (Figures 1B and S1). While some genes appeared to be used preferentially within each sample (Figure S1), there was no discernable pattern across all donors of longitudinal enrichment of particular genes. Similarly, the length distributions for the third heavy chain complementarity-determining region (CDRH3) remained relatively unchanged throughout infection for each donor (p > 0.1 for all CDRH3 lengths in all samples, *Z* test with Benjamini-Hochberg correction) (Figure 1C). Taken together, these results indicated that, while systems-level repertoire features were conserved over time, antibody sequence retention over the course of HIV-1 infection was low; rather, each donor was associated with virtually non-overlapping repertoires at the three different time points.

### Identification of Public Antibody Clonotypes in HIV-1 Infection

**Figure 2:**
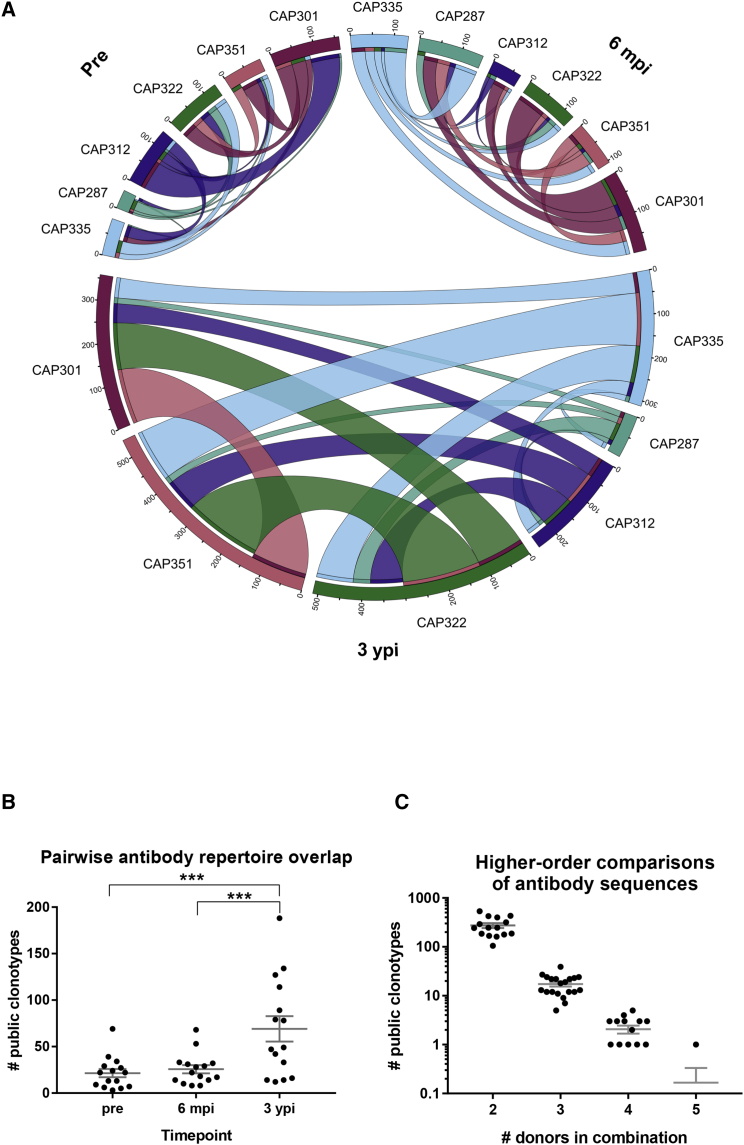
Identification of Public Antibody Clonotypes after Infection with HIV-1 (A) Clonal overlap between pre-infection (top left), 6 mpi (top right), and 3 ypi (bottom) samples. The width of the curved bands connecting each pair of samples is proportional to the numbers (tick labels) of antibody clonotypes shared by that donor pair at the given time point. (B) For each of the three time points (x axis), the numbers of public antibody clonotypes (y axis) are plotted for each pair (dots) of donors. ^∗∗∗^p < 0.001. Error bars: mean ± SEM. (C) For all combinations (dots) of two, three, four, and five donors (x axis), the corresponding numbers of public antibody clonotypes (y axis) across all time points are shown. Error bars: mean ± SEM.

Since recent studies have indicated the presence of public antibody clonotypes shared among individuals, including in the antigen-directed response (Jackson et al., 2014, Parameswaran et al., 2013), we set out to explore the presence of public antibody sequences in the case of HIV-1 infection. To identify public antibodies, we performed clonal family assignment on all samples from all donors simultaneously (STAR Methods). As expected, this approach resulted in most clonotypes consisting of sequences from single donors. However, we also identified a number of clonotypes with sequences from multiple donors (Figure 2), which we designated public clonotypes. Although these sequence groups are not technically biological clones (as they were derived from multiple individuals and thus are not derived from a single B cell), public clonotypes were defined as groups of sequences with the same V_H_ gene, the same J_H_ gene, the same junction length, and CDRH3 amino acid sequences of high identity between donors (STAR Methods).

We analyzed the antibody sequences using a range of junction region identity thresholds during the clonal assignment procedure (Figure S3A). Of note, public clonotypes were identified for all threshold values, including at 100% junction identity. Although identities between members of known bNAb lineages can be as low as ∼30% (Table S4), for further analysis we selected a conservative threshold of 70%, in which all members of a clonotype would have at least 70% junction region identity to all other members of the clonotype. This threshold aimed at allowing reasonable inclusion of intra-clonal evolution without allowing highly divergent sequences from different donors to be grouped together.

Comparison of the antibody repertoires between all pairs of donors revealed that public antibody clonotypes existed at each of the pre-infection, 6 mpi, and 3 ypi time points (Figure 2A). Intriguingly, at the 70% junction identity threshold used for this analysis, the number of public clonotypes significantly increased at 3 ypi (average number of public clonotypes from pairwise comparisons of 69.1) relative to pre-infection (average number of public clonotypes from pairwise comparisons of 21.5) (p < 0.0001, linear mixed effects model) (Figures 2A and 2B). Although the difference between the numbers of public clonotypes at pre-infection and 6 mpi was not significant (p = 0.69, linear mixed effects model), the difference between 6 mpi and 3 ypi was also significant (p = 0.0002, linear mixed effects model). These public clonotypes were not restricted to germline sequences and exhibited a wide distribution of V-gene deviation from germline (Figure S2).

To determine whether public clonotypes could be identified within larger subsets of donors, we compared the antibody repertoires for all combinations of two to six donors for all three time points (Figure 2C). Smaller numbers of clonotypes were detected as the number of donors between whom a clonotype was shared increased, but 27 clonotypes shared by at least four of the six donors also were identified (Figures 2C, S3B, and S3C). Interestingly, public antibodies present 3 ypi were found predominantly only after infection, with at most ∼7.5% of 3 ypi antibodies shared by any pair of donors present in the pre-infection repertoire of either donor (Figure S3D). Taken together, these results suggested that a non-negligible fraction of public antibodies appeared to emerge after HIV-1 infection (Figures S3D and S3E) alongside the highly diverse private antibody response.

### Confirmation of HIV-1 Reactivity of a Public Antibody Clonotype

**Figure 3:**
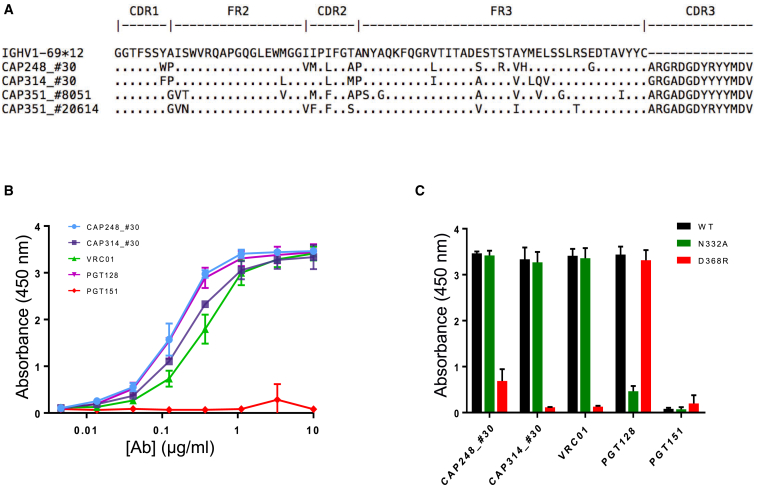
Characterization of a Public HIV-Reactive Antibody Clonotype Shared by Three HIV-Infected Donors (A) Multiple sequence alignment of the CDR1-CDR3 regions of the heavy chain sequences from a three-donor public clonotype. Included are antibodies CAP248_#30 and CAP314_#30, as well as representative CAP351 antibodies CAP351_#8051 and CAP351_#20614, along with *IGHV1-69^∗^12*, a top germline allele assignment for all antibodies shown. Dots within the V-gene show identity to germline, while letters show mutations from germline. (B) ELISA binding of CAP248_#30 and CAP314_#30 to ConC gp120 at increasing antibody concentrations (x axis), with antibodies VRC01 and PGT128 as positive controls, and PGT151 as a negative control. Error bars: mean ± SD. (C) ELISA binding at a concentration of 1.11 μg/mL of antibodies CAP248_#30 and CAP314_#30 to a wild-type ConC gp120 protein, ConC gp120 with a N332A mutation, and ConC gp120 with a D368R mutation. Control antibodies are VRC01 (D368R sensitive), PGT128 (N332A sensitive), and PGT151 (negative control). Error bars: mean ± SD.

We performed paired heavy-light chain sequencing of the antigen-specific repertoire of two additional donors, CAP248 and CAP314 (STAR Methods, Table S1), to explore the potential of public antibodies to be HIV-1 reactive. Comparison of these sequences with the sequences from each of the other six CAPRISA donors revealed a large public clonotype between CAP248, CAP314, and CAP351 (Figure 3). Examples of representative antibodies from this public clonotype, including CAP351 CDRH3 sequences within a 1–2 amino acid difference from each of antibodies CAP248_#30 and CAP314_#30, are shown in Figure 3A. The maximum junction difference among these sequences (Figures 3A and S3F) was three amino acids, or ∼81% identity, for CAP248_#30 and CAP314_#30. In addition to the high CDRH3 identity, 4 of 13 somatic mutation changes from the *IGHV1-69* germline gene in the CAP248 antibody were identical in one of the representative CAP351 antibodies. Remarkably, CAP314_#30 and CAP248_#30 each used the same light chain germline gene, *IGKV1-27*, with CDRL3 sequences differing by just one amino acid (Figure S3F). As paired heavy and light chain sequence information was available for CAP248_#30 and CAP314_#30, we produced these antibodies as recombinant immunoglobulin G proteins and tested their reactivity against HIV-1. The two antibodies bound Consensus C (ConC) gp120 (Figure 3B) and neutralized tier 1 viruses MN.3 and MW965 but did not neutralize tier 2 viruses (Figure S3G). Epitope mapping revealed that antigen binding by both antibodies was affected by the D368R mutation but not by the N332A mutation (Figure 3C), potentially suggesting a CD4-binding site epitope specificity. Overall, these results demonstrate that public HIV-reactive antibodies can emerge during the course of natural HIV-1 infection.

### Existence of Public Antibody Clonotypes with High Sequence Identity to Known HIV-1 Antibodies

**Figure 4:**
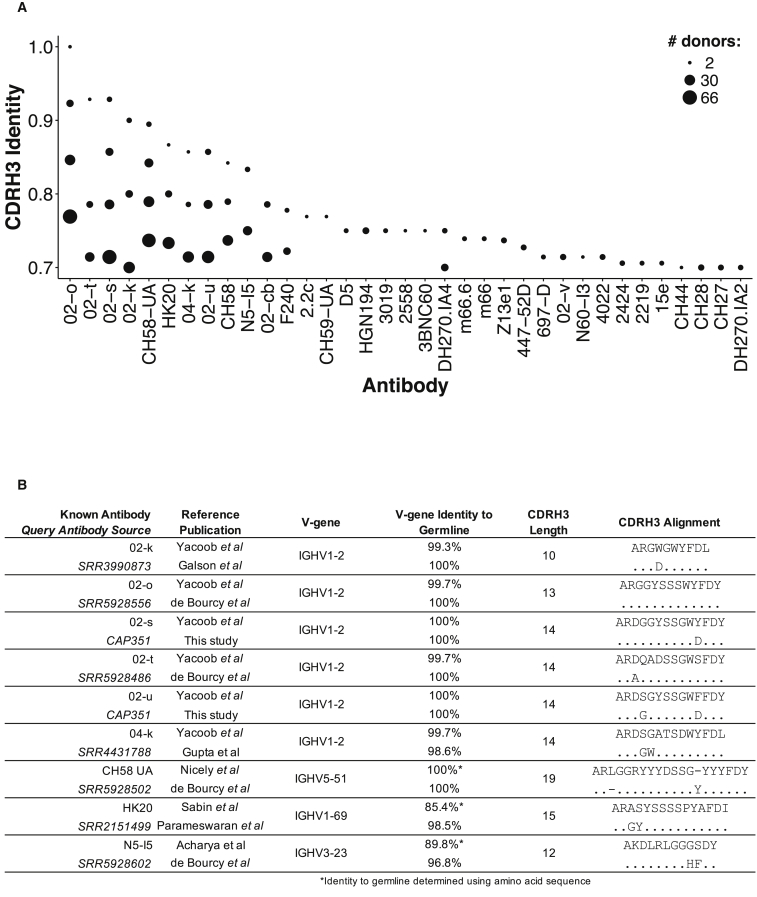
Comparison of Published Antibody Repertoires to Known HIV-Reactive Antibody Sequences (A) Number of donors (size of dots) with antibody heavy chain sequences with identical V-gene assignment, signature sequence features, and CDRH3 identity (y axis) of at least 70% to a set of known HIV-reactive antibodies (x axis). (B) Antibody heavy chain sequences with identical V-gene assignment, signature sequence features, and CDRH3 distance of at most two amino acids. For each pair of known/query antibody sequences, shown are the source dataset, references, V-gene assignment, percentage identity to germline, and CDRH3 length and alignment. V-gene deviation from germline for sequences obtained from the PDB was determined from amino acid sequence using IMGT/DomainGapAlign (Ehrenmann and Lefranc, 2011).

In general, while HIV-1 reactivity for an antibody cannot be determined solely from its sequence, it is informative to determine whether antibodies that are similar to known HIV-reactive sequences exist at the population level, as a way to assess the uniqueness of the space of antibody HIV-1 reactivity. We compared over 18 million heavy chain antibody sequences from approximately 250 publicly available next-generation sequencing samples, as well as from the samples presented in this study, to the heavy chain sequences of known HIV-reactive antibodies. To that end, we collected and curated a set of heavy chain sequences from HIV-reactive antibodies found in the PDB, CATNAP (Compile, Analyze and Tally NAb Panels) (Yoon et al., 2015) and literature (Yacoob et al., 2016) (STAR Methods). The sequencing data were derived from infection, vaccination, and autoimmunity studies, as well as from CAPRISA samples sequenced as part of this study. We then compared the CDRH3 amino acid sequences and V-gene assignments from all sequences in each sample with CDRH3 sequences and V-gene assignments encoding HIV-reactive antibodies (Figure 4). Specific sequence features known to be important for HIV-1 recognition, such as a requisite tryptophan in the fifth position preceding the framework 4 region for 3BNC60 (Scheid et al., 2011) and gp120-binding putative VRC01 precursors (Yacoob et al., 2016), were included as additional constraints in the comparison. As expected, many observed sequences had low identity to known HIV-1 antibodies. However, 35 known HIV-reactive antibodies had matches with at least 70% CDRH3 identity, in addition to matching V-gene and sequence feature requirements, to antibodies in the examined samples (Figure 4A). With these search parameters, as many as ∼70 donors were matched to a given HIV-reactive antibody. In addition, nine antibodies had matches within a difference of no more than two amino acids in the CDRH3 region, including an exact match for antibody 02-o (Yacoob et al., 2016) (Figure 4B). The nine known HIV-reactive antibodies (Figure 4B) included antibodies with extra-neutralization functions (Acharya et al., 2014), weakly neutralizing antibodies (Sabin et al., 2010), putative vaccine-elicited antibody precursors (Nicely et al., 2015), and putative bNAb precursors (Yacoob et al., 2016), and spanned a range of CDRH3 lengths from 10 to 19 amino acids. These results suggest that sequences within a short distance from HIV-reactive antibodies, including some that could be possible vaccine templates, exist at the population level.

## Discussion

Here, we interrogated the antibody heavy chain repertoires of multiple HIV-infected donors at multiple time points through next-generation sequencing and systems immunology analysis. Antibody sequence repertoires in chronic HIV-1 infection appeared to diverge from their pre-infection repertoires. While we cannot exclude sampling depth as a possible explanation for the observed repertoire turnover, virus-induced turnover or a more general property of B cell repertoire dynamics could also explain this observation.

The discovery of public antibody clonotypes in the setting of HIV-1 infection is intriguing, especially given the rapid viral evolution that occurs during HIV-1 infection. While previous studies (Parameswaran et al., 2013) have observed public antibodies following acute viral infections, these antigens are far less diverse than HIV-1. It is plausible that some of the identified public antibody clonotypes are not HIV specific. In particular, existence of public clonotypes with affinity-matured sequences in pre-infection repertoires (Figure S2) could indicate shared modes of recognition at the antibody sequence level in response to common, previously encountered antigens. However, public clonotypes that are HIV reactive also exist (Figure 3). Furthermore, public clonotypes with high sequence identity to known HIV-reactive antibodies also were identified in a variety of immune settings, including in HIV-naive individuals (Figure 4). Our analysis identified public clonotypes for potential HIV-1 bNAb precursors but less frequently for public mature bNAbs (Figure 4), suggesting the possibility that bNAbs may evolve from virtually identical recombination events that subsequently take divergent evolutionary pathways in different individuals. Taken together, our findings suggest that a number of HIV-reactive antibody sequences may be readily accessible in multiple individuals, or even at the population level. Future vaccine development strategies may therefore benefit from specifically avoiding immunogen engagement with non-neutralizing public antibody clonotypes, such as the three-donor *IGHV1-69* public clonotype characterized here, and aim to target engagement of bNAb or bNAb-precursor public clonotypes.

The complexity of the humoral response to HIV-1 infection warrants large-scale analyses, such as those described here. Future juxtaposition of the antibody repertoires of broad and weak neutralizers, and from before and after infection or vaccination, could prove to be critical for identifying key correlates of protection and for iterative improvement of vaccine strategies. Such vaccine strategies could seek to recapitulate repertoire features observed to be conducive to protection or aim to interact with specific and common features of the antibody repertoire, such as public sequences (Crowe and Koff, 2015). Further exploration of these possibilities with larger infection cohorts representative of global HIV-1 infection diversity is therefore needed.

## STAR★Methods

### Key Resources Table

**Table:** 

REAGENT or RESOURCE	SOURCE	IDENTIFIER
**Antibodies**		

VRC01	Mascola/NIH AIDS Reagent	GU980702/GU980703
PGT128	IAVI/NIH AIDS Reagent	JN201917/JN201900
PGT151	Hahn/Salazar	KJ700290/KJ700282
CD14-FITC (HCD14)	BioLegend	Cat # 325604
CD3-FITC (UCHT1)	BioLegend	Cat # 300406
IgM-A488 (MHM-88)	BioLegend	Cat # 314534
IgD-A488 (IA6-2)	BioLegend	Cat # 348216
CD19-BV421 (HIB19)	BioLegend	Cat # 302234
CD19-PE (SJ25C1)	eBioscience	Cat # 12-0198-42
CD20-PECy7	BioLegend	Cat # 302312
CD20-BV711 (2H7)	BioLegend	Cat # 302341
CD38-PECy7	BioLegend	Cat # 303516
CD38-PerCPCy5.5 (HIT2)	BioLegend	Cat # 303522
CD27-BV510 (O323)	BioLegend	Cat # 302836
IgA-FITC (IS11-8E10)	Miltenyi	Cat # 130-093-071

**Bacterial and Virus Strains**		

MN.3	Montefiori/Korber	HM215430.1
MW965	Hahn	U08455.1

**Biological Samples**		

PBMC of CAP301, Pre-infection	CAPRISA / University of KwaZulu Natal / NICD / University of the Witwatersrand	N/A
PBMC of CAP301, 6mpi	CAPRISA / University of KwaZulu Natal / NICD / University of the Witwatersrand	N/A
PBMC of CAP301, 3ypi	CAPRISA / University of KwaZulu Natal / NICD / University of the Witwatersrand	N/A
PBMC of CAP351, Pre-infection	CAPRISA / University of KwaZulu Natal / NICD / University of the Witwatersrand	N/A
PBMC of CAP351, 6mpi	CAPRISA / University of KwaZulu Natal / NICD / University of the Witwatersrand	NA
PBMC of CAP351, 3ypi	CAPRISA / University of KwaZulu Natal / NICD / University of the Witwatersrand	N/A
PBMC of CAP335, Pre-infection	CAPRISA / University of KwaZulu Natal / NICD / University of the Witwatersrand	N/A
PBMC of CAP335, 6mpi	CAPRISA / University of KwaZulu Natal / NICD / University of the Witwatersrand	N/A
PBMC of CAP335, 3ypi	CAPRISA / University of KwaZulu Natal / NICD / University of the Witwatersrand	N/A
PBMC of CAP287, Pre-infection	CAPRISA / University of KwaZulu Natal / NICD / University of the Witwatersrand	N/A
PBMC of CAP287, 6mpi	CAPRISA / University of KwaZulu Natal / NICD / University of the Witwatersrand	N/A
PBMC of CAP287, 3ypi	CAPRISA / University of KwaZulu Natal / NICD / University of the Witwatersrand	N/A
PBMC of CAP312, Pre-infection	CAPRISA / University of KwaZulu Natal / NICD / University of the Witwatersrand	N/A
PBMC of CAP312, 6mpi	CAPRISA / University of KwaZulu Natal / NICD / University of the Witwatersrand	N/A
PBMC of CAP312, 3ypi	CAPRISA / University of KwaZulu Natal / NICD / University of the Witwatersrand	N/A
PBMC of CAP322, Pre-infection	CAPRISA / University of KwaZulu Natal / NICD / University of the Witwatersrand	N/A
PBMC of CAP322, 6mpi	CAPRISA / University of KwaZulu Natal / NICD / University of the Witwatersrand	N/A
PBMC of CAP322, 3ypi	CAPRISA / University of KwaZulu Natal / NICD / University of the Witwatersrand	N/A
PBMC of CAP314, 2ypi	CAPRISA / University of KwaZulu Natal / NICD / University of the Witwatersrand	N/A
PBMC of CAP248, 5.9ypi	CAPRISA / University of KwaZulu Natal / NICD / University of the Witwatersrand	N/A

**Chemicals, Peptides, and Recombinant Proteins**		

ConC gp120	Morris (NICD)	NA
CAP45 SOSIP.664	Montefiori/Morris (NICD)	DQ435682.1
CAP314_#30	ATRECA	CAP314_#30
CAP248_#30	ATRECA	CAP248_#30

**Critical Commercial Assays**		

Antigen-specific Single Cell Sorting for CAP314	Morris (NICD)	ConC gp120 WT and ConC N332A D368R gp120.
Antigen-specific Single Cell Sorting for CAP248	Morris (NICD)	CAP45 SOSIP.664
Paired Chain Sequencing	ATRECA	Immune repertoire capture® (IRC™) as described by DeFalco et al., 2018
RNeasy Mini Kit	QIAGEN	Cat #74104

**Deposited Data**		

Illumina MiSeq sequencing of CAPRISA donor B cell receptor transcripts	This Paper	BioProject PRJNA415492
Publicly Available BCR Sequencing Data	NCBI Short Reads Archive	SRR5928522; ERR875304; SRR5928591; SRR5928527; SRR5408028; SRR5928484; SRR5928504; SRR5928590; SRR030817; SRR1383465; SRR030816; SRR3099140; SRR1200519; SRR2905692; SRR5928502; SRR5928499; SRR5928602; SRR5928598; SRR5928596; SRR5928582; SRR5928581; SRR5928579; SRR5928572; SRR5928561; SRR5928549; SRR5928545; SRR5928544; SRR5928538; SRR5928537; SRR5928529; SRR5928523; SRR5928510; SRR5928509; SRR5928507; SRR5928488; SRR5928486; SRR5928482; SRR5928481; SRR5408029; SRR5408021; SRR4026031; SRR3458041; SRR1961400; SRR1383473; SRR1383453; SRR1200520; SRR5928601; SRR5928597; SRR5928595; SRR5928532; SRR5928517; SRR5928515; SRR5928511; SRR5928490; SRR5928487; SRR5408026; SRR5408024; SRR5408022; SRR4431790; SRR4431769; SRR4431764; SRR5928493; SRR5928505; SRR5928548; SRR5928599; SRR5928492; SRR5928497; SRR1964711; SRR1383463; SRR4431772; SRR5408027; SRR5928528; SRR5928491; SRR5408020; SRR5928574; SRR5928500; SRR2905668; SRR5928541; SRR1964792; SRR5928576; SRR2151562; SRR5928503; SRR4026038; SRR5928485; SRR1964794; SRR5928513; SRR5928543; SRR5928501; SRR4431767; SRR5928520; SRR5928600; SRR4431777; SRR2905662; SRR3990893; SRR5408006; SRR5928498; SRR5928588; SRR5928526; SRR030813; SRR5928512; SRR4431782; SRR5928519; SRR5408023; SRR5408025; SRR1964710; SRR5928496; SRR3106470; SRR5928495; SRR3990903; SRR4431774; SRR5928563; SRR1383461; SRR5408014; SRR275668; SRR4026036; SRR5408005; SRR1383464; SRR030820; SRR4026032; SRR030822; SRR5928553; SRR1383459; SRR1964786; SRR4431771; SRR5928593; SRR5928518; SRR3099124; SRR5928546; SRR3990894; SRR5928547; SRR5928506; SRR5928589; SRR1383474; SRR5928483; SRR3990856; SRR2905704; SRR5408019; SRR3990832; SRR3992911; SRR1959703; ERR875299; SRR1964800; SRR2151247; SRR3099064; SRR2905698; SRR924016; SRR3088951; SRR5928554; SRR4431791; SRR4431786; SRR1383451; SRR1818729; SRR4431789; SRR5928556; SRR1383452; SRR1383326; SRR924017; SRR5928516; SRR5928533; SRR3990831; SRR3088952; SRR3099127; SRR5928531; SRR5928530; SRR4431788; SRR1964801; SRR1818730; SRR4026020; SRR2905710; SRR3088950; SRR275711; SRR2150420; SRR5928585; SRR3099179; SRR3990873; SRR800642; SRR2905709; SRR4431775; SRR277211; SRR4026035; SRR3992952; SRR4026015; ERR875291; SRR5408010; SRR3990841; SRR4431765; SRR2153265; SRR5408007; SRR1383448; SRR3992920; SRR5408008; SRR3106439; SRR5928562; SRR3106524; SRR1168789; SRR2153058; SRR4431793; SRR4026022; SRR5928489; SRR1964712; SRR3990851; SRR1964713; SRR3350724; SRR3106458; SRR5928550; SRR3099142; SRR2905656; SRR1818726; SRR2905674; SRR654169; SRR4431780; SRR4431787; SRR1383447; SRR4431768; SRR3106450; SRR2153061; SRR1964787; SRR3106475; SRR5928573; SRR5408011; SRR2153043; SRR2153066; SRR3106497; SRR3099175; SRR4431773; SRR2153072; SRR4431783; SRR5928571; SRR4431766; SRR4431779; SRR3990907; SRR2153250; SRR4026023; SRR3099144; SRR1964788; ERR875295; SRR3350720; SRR3099157; SRR3099071; SRR2153236; SRR4026027; SRR5928592; SRR4026008; SRR924015; SRR2151499; SRR4026033; SRR2151523; SRR2905685

**Software and Algorithms**		

pRESTO Version 0.5.3	(Vander Heiden et al., 2014)	https://presto.readthedocs.io/
Change-O Version 0.3.9	(Gupta et al., 2015)	http://changeo.readthedocs.io/
Mixcr Version 2.1.5	(Bolotin et al., 2015)	https://mixcr.readthedocs.io/
IgBLAST	(Ye et al., 2013)	https://ftp.ncbi.nih.gov/blast/executables/igblast/release/LATEST/
BioEdit	(Hall, 1999)	http://www.mbio.ncsu.edu/BioEdit/bioedit.html
PRISM 7	GraphPad Software	https://www.graphpad.com/scientific-software/prism/
IMGT	(Lefranc et al., 2015)	http://imgt.org
lme4	(Douglas et al., 2015)	https://cran.r-project.org/web/packages/lme4/index.html
lmerTest	(Kuznetsova et al., 2014)	https://cran.r-project.org/web/packages/lmerTest/index.html
editdistance Python library		https://github.com/aflc/editdistance
ANARCI	(Dunbar and Deane, 2016)	http://opig.stats.ox.ac.uk/webapps/sabdab-sabpred/ANARCI.php

### Contact for Reagent and Resource Sharing

Further information and requests for resources and reagents should be directed to the Lead Contact, Ivelin Georgiev (ivelin.georgiev@vanderbilt.edu).

### Experimental Models and Subject Details

Participants in the CAPRISA study with stored peripheral blood mononuclear cells (PBMCs) from pre-infection and post-infection time points were selected for this study. The CAPRISA 002 Acute Infection study was approved by the ethics committees of the University of KwaZulu-Natal (E013/04), the University of Cape Town (025/2004) and the University of the Witwatersrand (MM040202). All 8 donors provided written consent for the use of stored samples for HIV-related research projects. See Table S1 for further details. Gender and age of the donors was not a consideration in this study.

### Method Details

#### Library Preparation and Repertoire Sequencing

For sequencing of global antibody repertoires, total RNA was extracted from PBMCs and an RT-PCR reaction was performed using a primer mix (BIOMED2 to framework 1 and framework 4) designed to amplify all heavy-chain antibody variable gene regions in an unbiased fashion. The resulting amplicons were purified, and agarose gel electrophoresis was used to confirm complete primer removal and appropriate amplicon size. The amplicon sample was quantified prior to submission to the Vanderbilt Technologies for Advanced Genomics (VANTAGE) core for paired-end (2 x 300 bp) Illumina MiSeq sequencing. The 18 samples from 6 donors were multiplexed across 3 MiSeq runs. FASTQ files from Illumina MiSeq sequencing served as the main input for subsequent data analysis. As a control, a second sequencing run was conducted with all 18 samples on one chip (Figures S4A and S4B), from which we identified ∼0.55 million V(D)J sequences across the 18 samples. Public sequences from run 1 were frequently recovered in the second run (Figure S4B).

#### Sequencing Data Preprocessing and Clonal Analysis

Preprocessing was carried out using pRESTO (Vander Heiden et al., 2014) as follows: *1)* Paired-end reads were interleaved and reads with a mean Phred quality score below 20 were removed. *2)* Orientation of sequences was corrected to the forward orientation (V to J) as necessary. *3)* V-region primers were masked and C-region primers were cut. Sequences with no match to primers were discarded. *4)* Duplicate sequences were removed and a duplication count of each sequence was annotated. *5)* All sequences with duplication count of 1 were removed. A wide distribution of duplication counts was observed (Figure S4C). *6)* Each sequence was annotated for V, D, and J gene usage using IgBLAST (Ye et al., 2013), using reference sequences from IMGT (Lefranc et al., 2015). After removing non-functional sequences and sequences with CDRH3 lengths of under 5 amino acids, clonal clustering of all 1316148 V(D)J sequences from all donors was performed using Change-O (Gupta et al., 2015) (Figure S4D). Functional V_H_ V(D)J sequences were assigned to clonal groups by first grouping sequences based on common *IGHV* gene annotation, *IGHJ* gene annotation and junction region lengths. *IGHV* and *IGHJ* gene annotations for each group of sequences were determined by the first gene assignment of gene assignments within each junction length. Within these larger groups, sequences differing from one another by a threshold distance within the junction region were defined as clonotypes by complete-linkage clustering. Distance was determined using an amino acid Hamming distance normalized to the length of the junction. We used amino acid distance during clustering because this is the determinant of antibody molecular recognition; however, nucleotide and amino acid identities were generally very similar (Figure S4E).

#### Single Cell Sorting

Paired chain antibody sequencing for CAP248 and CAP314 was carried out by Atreca (Redwood City, CA) on IgG cells sorted into microtiter plates at one cell per well by FACS. Briefly, cryopreserved PBMCs were stained with the following antibodies: CD14-FITC (HCD14), CD3-FITC (UCHT1), IgM-A488 (MHM-88), IgD-A488 (IA6-2), CD20-PECy7 or CD20-BV711 (2H7), CD38-PECy7 or CD38-PerCPCy5.5 (HIT2), CD27-BV510 (O323) from BioLegend, CD19-BV421 (HIB19) from BioLegend or CD19-PE (SJ25C1) from eBiosciences, and IgA-FITC (IS11-8E10) from Miltenyi. For CAP248 PBMCs, antigen-specific cells were isolated using CAP45 SOSIP.664 trimeric protein. For CAP314 PBMCs, antigen-specific cells were isolated using ConC gp120. The sorted antigen-specific B cells were cultured for 4 days in IMDM medium (Invitrogen) in the presence of FBS, Normocin, IL-2 (PeproTech), IL-21 (PeproTech), rCD40 ligand (R&D Systems), and His-Tag antibodies (R&D Systems), prior to single cell sequencing.

#### Paired Chain Antibody Sequencing

Generation of barcoded cDNA, PCR amplification, and next-generation sequencing of paired IgG heavy & light chains were performed as described in (DeFalco et al., 2018), with the following modifications: desthiobiotinylated oligo (dT) and Maxima H Minus RT (Fisher Scientific Company) were used for reverse transcription, DynaBeads™ MyOne™ Streptavidin C1 (Life Technologies) was used to isolate desthiobiotinylated cDNA, PCR amplicon concentrations were determined using qPCR (KAPA SYBR® FAST qPCR Kit for Illumina, Kapabiosystems), and amplicons were sequenced on an Illumina MiSeq instrument.

#### Barcode Assignment, Sequence Assembly, Assignment of V(D)J and Identification of Mutations in Paired Chain Sequencing

FASTQ output files were grouped and parsed into separate FASTQ files on the basis of their compound ID (plate-ID + well-ID). We used Atreca proprietary software to assemble paired end reads into consensus sequences, requiring a minimum coverage of 30 reads for each heavy and each light chain assembly. Wells with more than one contig for a given chain type were rejected from consideration unless one of the contigs included at least 90% of the reads. V(D)J assignment and mutation identification was performed using IgBLAST (Ye et al., 2013). Allele assignments (Figures 3A and S3F) were determined using IMGT/V-Quest (Brochet et al., 2008).

#### Comparison of Antibody Repertoire Sequencing Data to Known HIV-Reactive Antibodies

A list of HIV-reactive antibodies was curated manually from the Protein Databank, CATNAP (Yoon et al., 2015), and literature (Yacoob et al., 2016). CDRH3 sequence and V-gene usage of each antibody was determined using ANARCI (Dunbar and Deane, 2016) with IMGT numbering. Publicly available antibody sequencing datasets collected from the Short Read Archive and the European Nucleotide Archive, in addition to the samples presented in this study, were processed via MiXCR (Bolotin et al., 2015). Briefly, reads in each sample were aligned with the *mixcr align* command and clustered by junction sequence and V-gene assignment using the *mixcr assemble* command with the *–OseparateByV=true* flag. First and last residues of the junction were trimmed to match the IMGT CDRH3 definition, and the first V-gene assignment in the alignment score-ranked list of possible gene assignments was used for comparison to the list of HIV-reactive antibodies. Within each sample, only sequences with a matching V-gene assignment and CDRH3 length to each HIV-1 antibody were compared for CDRH3 identity. Sequence identities were calculated using the *editdistance* library in Python and normalized to the length of the CDRH3 sequence. For antibodies 3BNC60, 02-cb, 02-o, 02-s, 02-u, 02-k, 02-t, 02-v, and 04-k, the requirement of a tryptophan in the fifth position preceding the framework 4 region also was imposed. Each accession number used in this study was manually annotated for donor ID in order to count the number of donors with matches to each HIV-reactive antibody (Figure 4). To retrieve identity to germline of MiXCR-processed data, alignments were exported using the *exportAlignments* command including the *-vBestIdentityPercent* flag. This calculates the fraction of matching nucleotides with the germline V-gene divided by the alignment length, which varied among publicly available samples due to differing sample preparation methods and sequencing strategies.

#### Neutralization Fingerprinting

Neutralization fingerprinting analysis of donor sera was performed as described previously (Doria-Rose et al., 2017). Briefly, epitope-specific neutralization fingerprints were constructed for ten antibody specificities against a panel of 21 diverse HIV-1 strains (Georgiev et al., 2013). For each serum, the serum-virus neutralization data against the same panel of HIV-1 strains was compared to the epitope-specific antibody fingerprints, in order to estimate the relative contribution of each of these reference antibody specificities to the polyclonal serum neutralization. For each serum, the estimated neutralization contributions by each of the ten reference antibody specificities were reported on a scale of 0 to 1, with all specificities adding up to 1.

### Quantification and Statistical Analysis

To determine statistical significance of pairwise donor overlap across timepoints (Figure 2), a linear mixed effects model was fit with timepoints designated as fixed effects and donors treated as random effects, thereby accounting for any correlation resulting from each donor being part of 5 pairwise comparisons per timepoint. Briefly, each donor was represented by a 45-dimensional vector, with each dimension having a value of 1 or 0 based on if that donor was part of each of the 45 pairwise comparisons that occurred across the 3 timepoints. The linear mixed effects model was then fit using the *lmer* function from the lme4 package (Douglas et al., 2015) in R. P values were determined using the lmerTest package (Kuznetsova et al., 2014) in R. Other statistical tests were performed using R version 3.4.1.

### Data and Software Availability

Sequencing data of global B-cell repertoires generated in this study have been deposited for public access under BioProject PRJNA415492.

## Supporting Citations

The following references appear in the Supplemental Information: Cheadle et al., 2003, Crooks et al., 2004, Doria-Rose et al., 2015, Shugay et al., 2015.

## Supplemental Information

Document S1. Figures S1–S4 and Tables S1–S4

Document S2. Article plus Supplemental Information

## References

[bib1] Acharya P., Tolbert W.D., Gohain N., Wu X., Yu L., Liu T., Huang W., Huang C.C., Kwon Y.D., Louder R.K. (2014). Structural definition of an antibody-dependent cellular cytotoxicity response implicated in reduced risk for HIV-1 infection. J. Virol..

[bib2] Ackerman M.E., Mikhailova A., Brown E.P., Dowell K.G., Walker B.D., Bailey-Kellogg C., Suscovich T.J., Alter G. (2016). Polyfunctional HIV-specific antibody responses are associated with spontaneous HIV control. PLoS Pathog..

[bib3] Arentz G., Thurgood L.A., Lindop R., Chataway T.K., Gordon T.P. (2012). Secreted human Ro52 autoantibody proteomes express a restricted set of public clonotypes. J. Autoimmun..

[bib4] Bolotin D.A., Poslavsky S., Mitrophanov I., Shugay M., Mamedov I.Z., Putintseva E.V., Chudakov D.M. (2015). MiXCR: software for comprehensive adaptive immunity profiling. Nat. Methods.

[bib5] Bonsignori M., Zhou T., Sheng Z., Chen L., Gao F., Joyce M.G., Ozorowski G., Chuang G.-Y., Schramm C.A., Wiehe K. (2016). Maturation pathway from germline to broad HIV-1 neutralizer of a CD4-mimic antibody. Cell.

[bib6] Brochet X., Lefranc M.P., Giudicelli V. (2008). IMGT/V-QUEST: the highly customized and integrated system for IG and TR standardized V-J and V-D-J sequence analysis. Nucleic Acids Res..

[bib7] Burton D.R., Mascola J.R. (2015). Antibody responses to envelope glycoproteins in HIV-1 infection. Nat. Immunol..

[bib8] Cheadle C., Vawter M.P., Freed W.J., Becker K.G. (2003). Analysis of microarray data using Z score transformation. J. Mol. Diagn..

[bib9] Corey L., Gilbert P.B., Tomaras G.D., Haynes B.F., Pantaleo G., Fauci A.S. (2015). Immune correlates of vaccine protection against HIV-1 acquisition. Sci. Transl. Med..

[bib10] Crooks G.E., Hon G., Chandonia J.M., Brenner S.E. (2004). WebLogo: a sequence logo generator. Genome Res..

[bib11] Crowe J.E., Koff W.C. (2015). Deciphering the human immunome. Expert Rev. Vaccines.

[bib12] DeFalco J., Harbell M., Manning-Bog A., Baia G., Scholz A., Millare B., Sumi M., Zhang D., Chu F., Dowd C. (2018). Non-progressing cancer patients have persistent B cell responses expressing shared antibody paratopes that target public tumor antigens. Clin. Immunol..

[bib13] De Milito A., Nilsson A., Titanji K., Thorstensson R., Reizenstein E., Narita M., Grutzmeier S., Sönnerborg A., Chiodi F. (2004). Mechanisms of hypergammaglobulinemia and impaired antigen-specific humoral immunity in HIV-1 infection. Blood.

[bib14] Doria-Rose N.A., Schramm C.A., Gorman J., Moore P.L., Bhiman J.N., DeKosky B.J., Ernandes M.J., Georgiev I.S., Kim H.J., Pancera M. (2014). Developmental pathway for potent V1V2-directed HIV-neutralizing antibodies. Nature.

[bib15] Doria-Rose N.A., Bhiman J.N., Roark R.S., Schramm C.A., Gorman J., Chuang G.Y., Pancera M., Cale E.M., Ernandes M.J., Louder M.K. (2015). New member of the V1V2-directed CAP256-VRC26 lineage that shows increased breadth and exceptional potency. J. Virol..

[bib16] Doria-Rose N.A., Altae-Tran H.R., Roark R.S., Schmidt S.D., Sutton M.S., Louder M.K., Chuang G.Y., Bailer R.T., Cortez V., Kong R. (2017). Mapping polyclonal HIV-1 antibody responses via next-generation neutralization fingerprinting. PLoS Pathog..

[bib17] Douglas B., Maechler M., Ben B., Walker S. (2015). Fitting linear mixed-effects models using lme4. J. Stat. Softw..

[bib18] Dunbar J., Deane C.M. (2016). ANARCI: antigen receptor numbering and receptor classification. Bioinformatics.

[bib19] Ehrenmann F., Lefranc M.P. (2011). IMGT/DomainGapAlign: IMGT standardized analysis of amino acid sequences of variable, constant, and groove domains (IG, TR, MH, IgSF, MhSF). Cold Spring Harb. Protoc..

[bib20] Georgiev I.S., Doria-Rose N.A., Zhou T., Do Kwon Y., Staupe R.P., Moquin S., Chuang G.-Y., Louder M.K., Schmidt S.D., Altae-Tran H.R. (2013). Delineating antibody recognition in polyclonal sera from patterns of HIV-1 isolate neutralization. Science.

[bib21] Gupta N.T., Vander Heiden J.A., Uduman M., Gadala-Maria D., Yaari G., Kleinstein S.H. (2015). Change-O: a toolkit for analyzing large-scale B cell immunoglobulin repertoire sequencing data. Bioinformatics.

[bib22] Hall T.A. (1999). BioEdit: a user-friendly biological sequence alignment editor and analysis program for Windows 95/98/NT. Nucleic Acids Symp. Ser..

[bib23] Harrer T., Harrer E., Kalams S., Elbeik T., Staprans S.I., Feinberg M.B., Cao Y., Ho D.D., Yilma T., Caliendo A.M. (1996). Strong cytotoxic T cell and weak neutralizing antibody responses in a subset of persons with stable nonprogressing HIV type 1 infection. AIDS Res. Hum. Retroviruses.

[bib24] Henry Dunand C.J., Wilson P.C. (2015). Restricted, canonical, stereotyped and convergent immunoglobulin responses. Philos. Trans. R. Soc. Lond. B Biol. Sci..

[bib25] Horwitz J.A., Bar-On Y., Lu C.L., Fera D., Lockhart A.A.K., Lorenzi J.C.C., Nogueira L., Golijanin J., Scheid J.F., Seaman M.S. (2017). Non-neutralizing antibodies alter the course of HIV-1 infection in vivo. Cell.

[bib26] Huang J., Kang B.H., Ishida E., Zhou T., Griesman T., Sheng Z., Wu F., Doria-Rose N.A., Zhang B., McKee K. (2016). Identification of a CD4-binding-site antibody to HIV that evolved near-pan neutralization breadth. Immunity.

[bib27] Jackson K.J.L., Liu Y., Roskin K.M., Glanville J., Hoh R.A., Seo K., Marshall E.L., Gurley T.C., Moody M.A., Haynes B.F. (2014). Human responses to influenza vaccination show seroconversion signatures and convergent antibody rearrangements. Cell Host Microbe.

[bib28] Jardine J.G., Kulp D.W., Havenar-Daughton C., Sarkar A., Briney B., Sok D., Sesterhenn F., Ereno-Orbea J., Kalyuzhniy O., Deresa I. (2016). HIV-1 broadly neutralizing antibody precursor B cells revealed by germline-targeting immunogen. Science.

[bib29] Kaufmann D.E., Kavanagh D.G., Pereyra F., Zaunders J.J., Mackey E.W., Miura T., Palmer S., Brockman M., Rathod A., Piechocka-Trocha A. (2007). Upregulation of CTLA-4 by HIV-specific CD4+ T cells correlates with disease progression and defines a reversible immune dysfunction. Nat. Immunol..

[bib30] Kuznetsova, A., Brockhoff, P.B., and Christensen, R.H.B. (2014). Tests for random and fixed effects for linear mixed effect models (lmer objects of lme4 package). R package version 2.0-3. http://cran.r-project.org/package=lmerTest.

[bib31] Lefranc M.P., Giudicelli V., Duroux P., Jabado-Michaloud J., Folch G., Aouinti S., Carillon E., Duvergey H., Houles A., Paysan-Lafosse T. (2015). IMGT, the international ImMunoGeneTics information system 25 years on. Nucleic Acids Res..

[bib32] Liao H.-X., Lynch R., Zhou T., Gao F., Alam S.M., Boyd S.D., Fire A.Z., Roskin K.M., Schramm C.A., Zhang Z. (2013). Co-evolution of a broadly neutralizing HIV-1 antibody and founder virus. Nature.

[bib33] Nicely N.I., Wiehe K., Kepler T.B., Jaeger F.H., Dennison S.M., Rerks-Ngarm S., Nitayaphan S., Pitisuttithum P., Kaewkungwal J., Robb M.L. (2015). Structural analysis of the unmutated ancestor of the HIV-1 envelope V2 region antibody CH58 isolated from an RV144 vaccine efficacy trial vaccinee. EBioMedicine.

[bib34] Palmer B.E., Boritz E., Wilson C.C. (2004). Effects of sustained HIV-1 plasma viremia on HIV-1 gag-specific CD4+ T cell maturation and function. J. Immunol..

[bib35] Pancera M., Zhou T., Druz A., Georgiev I.S., Soto C., Gorman J., Huang J., Acharya P., Chuang G.-Y., Ofek G. (2014). Structure and immune recognition of trimeric pre-fusion HIV-1 Env. Nature.

[bib36] Parameswaran P., Liu Y., Roskin K.M., Jackson K.K.L., Dixit V.P., Lee J.-Y., Artiles K., Zompi S., Vargas M.J., Simen B.B. (2013). Convergent antibody signatures in human dengue. Cell Host Microbe.

[bib37] Pieper K., Tan J., Piccoli L., Foglierini M., Barbieri S., Chen Y., Silacci-Fregni C., Wolf T., Jarrossay D., Anderle M. (2017). Public antibodies to malaria antigens generated by two LAIR1 insertion modalities. Nature.

[bib38] Rademeyer C., Korber B., Seaman M.S., Giorgi E.E., Thebus R., Robles A., Sheward D.J., Wagh K., Garrity J., Carey B.R. (2016). Features of recently transmitted HIV-1 clade C viruses that impact antibody recognition: implications for active and passive immunization. PLoS Pathog..

[bib39] Rinaldo C., Huang X.L., Fan Z.F., Ding M., Beltz L., Logar A., Panicali D., Mazzara G., Liebmann J., Cottrill M. (1995). High levels of anti-human immunodeficiency virus type 1 (HIV-1) memory cytotoxic T-lymphocyte activity and low viral load are associated with lack of disease in HIV-1-infected long-term nonprogressors. J. Virol..

[bib40] Sabin C., Corti D., Buzon V., Seaman M.S., Hulsik D.L., Hinz A., Vanzetta F., Agatic G., Silacci C., Mainetti L. (2010). Crystal structure and size-dependent neutralization properties of HK20, a human monoclonal antibody binding to the highly conserved heptad repeat 1 of gp41. PLoS Pathog..

[bib41] Scheid J.F., Mouquet H., Ueberheide B., Diskin R., Klein F., Oliveira T.Y.K., Pietzsch J., Fenyo D., Abadir A., Velinzon K. (2011). Sequence and structural convergence of broad and potent HIV antibodies that mimic CD4 binding. Science.

[bib42] Shugay M., Bagaev D.V., Turchaninova M.A., Bolotin D.A., Britanova O.V., Putintseva E.V., Pogorelyy M.V., Nazarov V.I., Zvyagin I.V., Kirgizova V.I. (2015). VDJtools: unifying post-analysis of T cell receptor repertoires. PLoS Comput. Biol..

[bib43] Trück J., Ramasamy M.N., Galson J.D., Rance R., Parkhill J., Lunter G., Pollard A.J., Kelly D.F. (2015). Identification of antigen-specific B cell receptor sequences using public repertoire analysis. J. Immunol..

[bib44] Vander Heiden J.A., Yaari G., Uduman M., Stern J.N.H., O’Connor K.C., Hafler D.A., Vigneault F., Kleinstein S.H. (2014). PRESTO: a toolkit for processing high-throughput sequencing raw reads of lymphocyte receptor repertoires. Bioinformatics.

[bib45] Ward A.B., Wilson I.A. (2015). Insights into the trimeric HIV-1 envelope glycoprotein structure. Trends Biochem. Sci..

[bib46] Wu X., Zhou T., Zhu J., Zhang B., Georgiev I., Wang C., Chen X., Longo N.S., Louder M., McKee K. (2011). Focused evolution of HIV-1 neutralizing antibodies revealed by structures and deep sequencing. Science.

[bib47] Yacoob C., Pancera M., Vigdorovich V., Oliver B.G., Glenn J.A., Feng J., Sather D.N., McGuire A.T., Stamatatos L. (2016). Differences in allelic frequency and CDRH3 region limit the engagement of HIV Env immunogens by putative VRC01 neutralizing antibody precursors. Cell Rep..

[bib48] Ye J., Ma N., Madden T.L., Ostell J.M. (2013). IgBLAST: an immunoglobulin variable domain sequence analysis tool. Nucleic Acids Res..

[bib49] Yoon H., Macke J., West A.P., Foley B., Bjorkman P.J., Korber B., Yusim K. (2015). CATNAP: a tool to compile, analyze and tally neutralizing antibody panels. Nucleic Acids Res..

[bib50] Zhang R., Fichtenbaum C.J., Hildeman D.A., Lifson J.D., Chougnet C. (2004). CD40 ligand dysregulation in HIV infection: HIV glycoprotein 120 inhibits signaling cascades upstream of CD40 ligand transcription. J. Immunol..

[bib51] Zhou T., Lynch R.M., Chen L., Acharya P., Wu X., Doria-Rose N.A., Joyce M.G., Lingwood D., Soto C., Bailer R.T. (2015). Structural repertoire of HIV-1-neutralizing antibodies targeting the CD4 supersite in 14 donors. Cell.

