# Supplementary material for: Multi-Donor Longitudinal Antibody Repertoire Sequencing Reveals the Existence of Public Antibody Clonotypes in HIV-1 Infection
Source: Cell Host Microbe. 2018 Jun 13;23(6):845–854.e6. doi: 10.1016/j.chom.2018.05.001 (PMC6002606; doi:10.1016/j.chom.2018.05.001)
Supplement: Document S1. Figures S1–S4 and Tables S1–S4 [file mmc1.pdf]

Cell Host & Microbe, Volume *23*

Supplemental Information

Multi-Donor Longitudinal Antibody Repertoire Sequencing Reveals the Existence of Public Antibody Clonotypes in HIV-1 Infection

Ian Setliff, Wyatt J. McDonnell, Nagarajan Raju, Robin G. Bombardi, Amyn A. Murji, Cathrine Scheepers, Rutendo Ziki, Charissa Mynhardt, Bryan E. Shepherd, Alusha A. Mamchak, Nigel Garrett, Salim Abdool Karim, Simon A. Mallal, James E. Crowe Jr., Lynn Morris, and Ivelin S. Georgiev

Enriched, p<0.05
Enriched, p<0.10
Not enriched

***IGHV* gene enrichment within each sample**

	**CAP322**			**CAP351**			**CAP335**			**CAP312**			**CAP287**			**CAP301**		
	pre	6mpi	3ypi	pre	6mpi	3ypi	pre	6mpi	3ypi	pre	6mpi	3ypi	pre	6mpi	3ypi	pre	6mpi	3ypi
IGHV1-18	0.753	0.847	0.818	0.838	0.867	0.839	0.384	0.412	0.764	0.010	0.901	0.860	0.807	0.763	0.797	0.098	0.521	0.457
IGHV4-34	0.040	0.687	0.064	0.838	0.504	0.105	0.398	0.806	0.236	0.777	0.901	0.771	0.807	0.103	0.098	0.835	0.751	0.741
IGHV4-38-2	0.753	0.847	0.818	0.838	0.867	0.940	0.752	0.806	0.764	0.777	0.962	0.995	0.617	0.422	0.074	0.835	0.751	0.741
IGHV4-39	0.012	0.021	0.027	0.020	0.010	0.066	0.752	0.806	0.491	0.777	0.031	0.037	0.264	0.422	0.038	0.019	0.038	0.192
IGHV4-4	0.753	0.847	0.818	0.838	0.867	0.839	0.892	0.959	0.926	0.777	0.001	0.037	0.011	0.763	0.773	0.827	0.521	0.741
IGHV4-59	0.753	0.079	0.818	0.238	0.010	0.066	0.192	0.076	0.235	0.559	0.901	0.771	0.807	0.763	0.716	0.098	0.024	0.192

Figure S1, Related to Figure 1. Longitudinal analysis of gene usage enrichment. Clonotype abundance of each V gene was normalized by timepoint for each donor as described in (Cheadle et al., 2003). These standard scores were then tested with *Z*-tests, the *P* values of which were then adjusted using adaptive Benjamini-Hochberg correction for an FDR of 0.05. Genes with at least one $P < 0.1$ are shown, and all *P* values lower than, respectively, 0.10 and 0.05 after correction are colored in this panel.

A

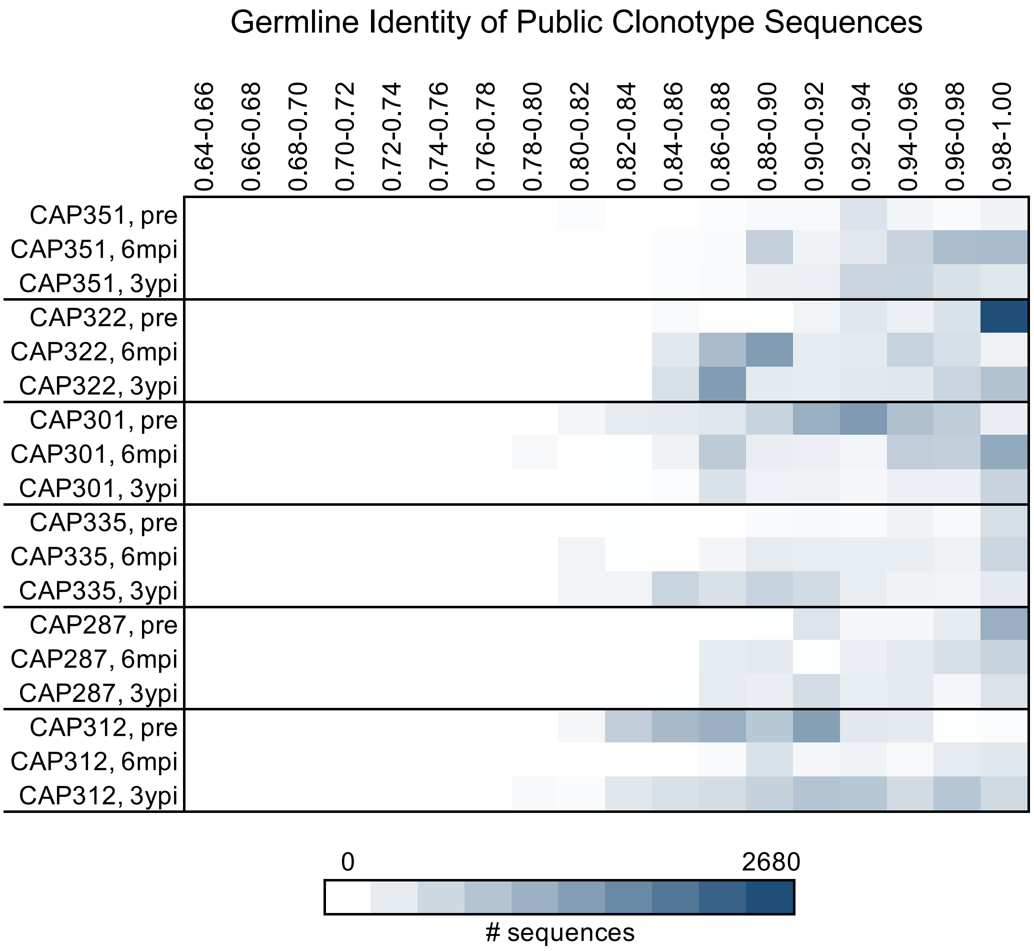

B

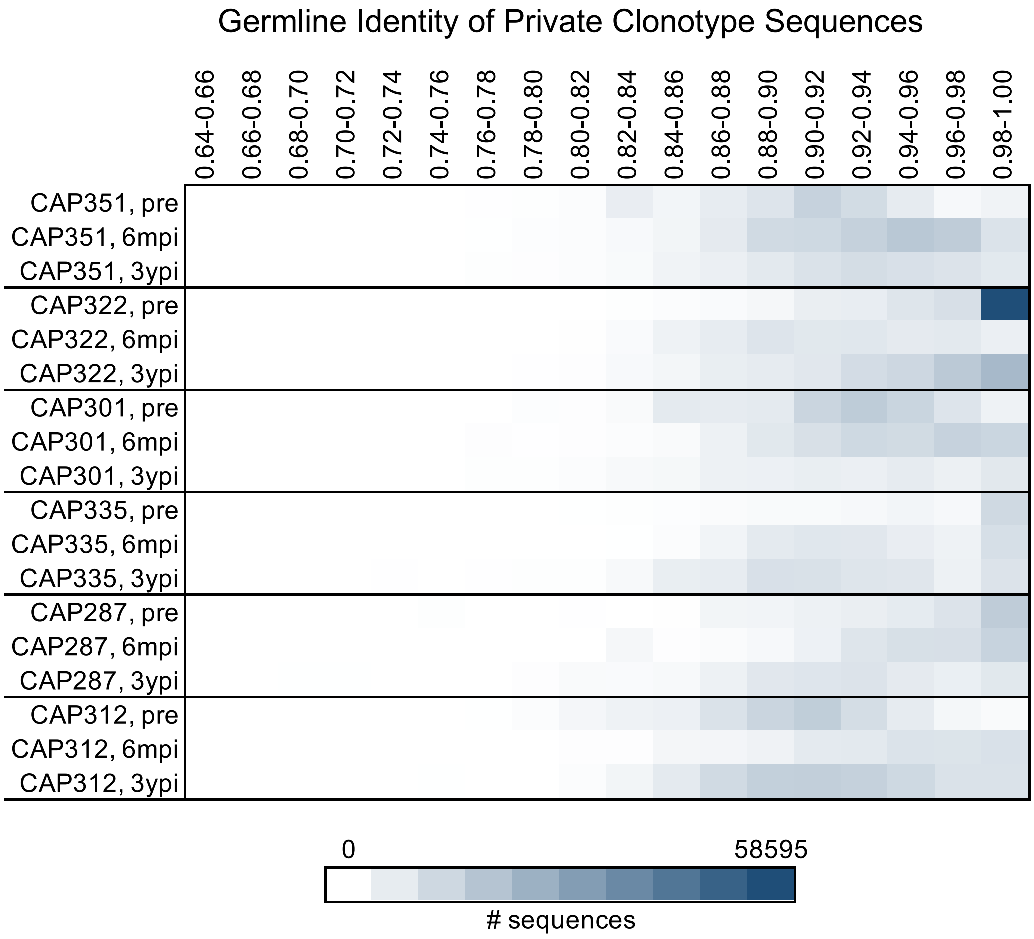

Figure S2, Related to Figure 2. Somatic hypermutation distributions of the public (A) and private (B) clonotypes for each donor (rows), binned (columns) by identity to germline in increments of 2%, with each bin being right-open, except for 0.98-1.00, which is inclusive of 1.00. Heatmap color intensity is proportional to the number of sequences in each bin, for each sample.

Figure S3

A

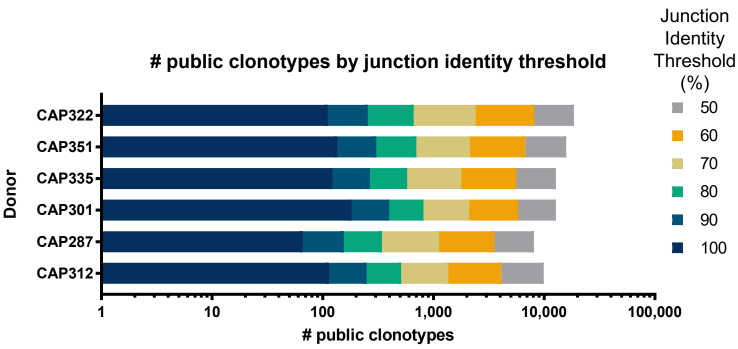

B

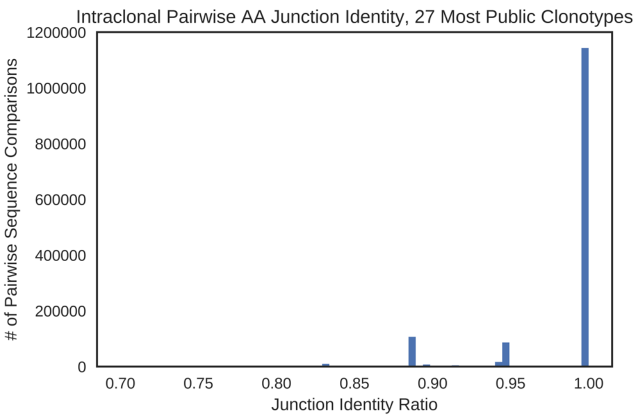

C

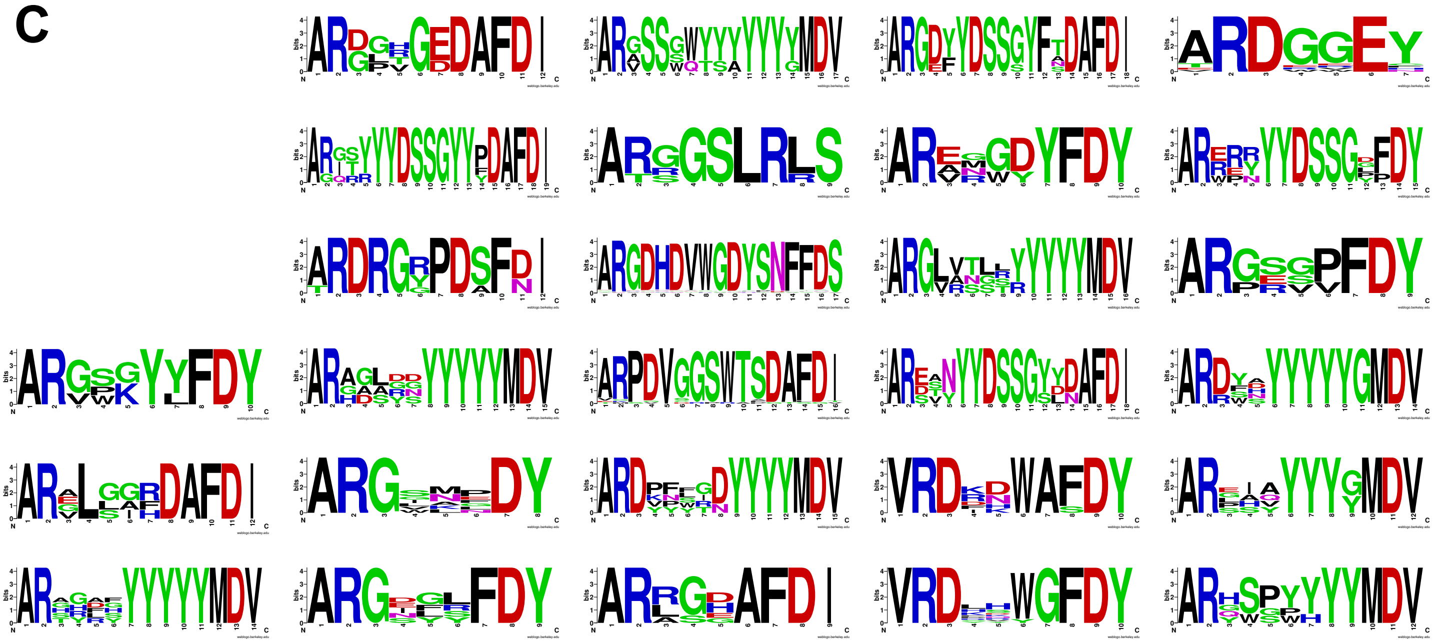

D

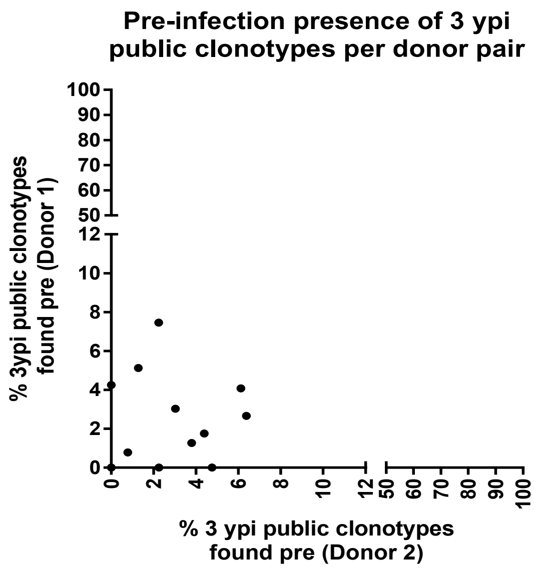

E

CAP322 — *P* = 1.000

3 ypi present pre-infection	Public (2+ donors): *Yes*	*No*
Yes	43	201
No	962	13714

CAP312 — *P* = 0.981

3 ypi present pre-infection	Public (2+ donors): *Yes*	*No*
Yes	2	10
No	418	8008

CAP351 — *P* = 0.841

3 ypi present pre-infection	*Yes*	*No*
Yes	22	256
No	894	12660

CAP301 — *P* = 0.978

3 ypi present pre-infection	*Yes*	*No*
Yes	19	112
No	591	5698

CAP335 — *P* = 0.747

3 ypi present pre-infection	*Yes*	*No*
Yes	5	55
No	465	6084

CAP287 — *P* = 0.813

3 ypi present pre-infection	*Yes*	*No*
Yes	1	11
No	146	2056

F

```
                        FR1                CDR1         FR2          CDR2                    FR3                           CDR3        FR4
               |-------------------------|------|-----------------|------|--------------------------------------|--------------|---------|

IGHV1-69*12    QVQLVQSGAEVKKPGSSVKVSCKASGGTFSSYAISWVRQAPGQGLEWMGGIIPIFGTANYAQKFQGRVTITADESTSTAYMELSSLRSEDTAVYYC-------------------------
CAP248_#30     ...............................WP.................VM.L..AP.........L.....S..R.VH........G.......ARGRDGDYRYYMDVWGEGTPVTVSS
CAP314_#30     ......................R........FP..............L.....L..MP.........I.....A....V.LQV.............GRGADGDYYYYMDVWGDGTTVIVSA

                        FR1                CDR1         FR2      CDR2                  FR3                    CDR3       FR4
               |-------------------------|----|-----------------|--|----------------------------------|---------|--------|

IGKV1-27*01    DIQMTQSPSSLSASVGDRVTITCRASQGISNYLAWYQQKPGKVPKLLIYAASTLQSGVPSRFSGSGSGTDFTLTISSLQPEDVATYYC-------------------
CAP314_#30.lc  .................K...........RH..................G...H...............H............F.....QKYDSGPRTFGPGTKVDFK
CAP248_#30.lc  ..............................T..................G...................Y............F.....QKYDSAPRTFGQGTNVDLK
```

G

Antibody	Pseudovirus: MN.3	MW965
CAP248_#30	0.1086	0.0133
CAP314_#30	0.1651	0.0278

Figure S3, Related to Figure 2 and Figure 3. Analysis of public antibody clonotypes in HIV-1 infection.

a. Number of public clonotypes (x-axis) for different junction identity clustering thresholds (colors) for each donor (y-axis).
b. Number of pairwise sequence comparisons (y-axis) with given amino acid junction identities (x-axis) between pairs of members within each of the 27 most public clonotypes.
c. Logo plots of 27 public clonotypes identified in 4 or more of the 6 CAPRISA donors whose global antibody repertoires were sequenced. Plots were generated using unique donor-deduplicated CDRH3 sequences and WebLogo as described in (Crooks et al., 2004). Briefly, the height of each amino acid is proportional to sequence conservation at that position, and the amino acids are colored by physicochemical properties. Member CDRH3 sequences in a given public clonotype sequence were deduplicated to unique CDRH3s and then used to generate a consensus sequence. These consensus sequences were manually curated to ensure that each sequence was present in its donor, and then used to build the final inter-donor consensus sequences displayed here.
d. Retention of public clonotypes during chronic infection with HIV-1. For each pair of donors (axes), shown is the fraction of shared clonotypes at 3ypi that were also found pre-infection.
e. Contingency tables testing for presence of 3ypi public/private clonotypes (x-axis) in pre-infection repertoires (y-axis) of each donor; *P* values are from Fisher's exact test with simulated *P* value from 1000 bootstraps.
f. Multiple sequence alignment of the heavy and light chain sequences from antibodies CAP248_#30 and CAP314_#30, along with the respective germline genes. Dots within the V-gene indicate no changes from germline, while letters show mutations from germline.
g. Neutralization data for antibodies CAP248_#30 and CAP314_#30. Displayed are the IC_{50} values of each antibody against tier 1 viruses MN.3 and MW965.

Figure S4

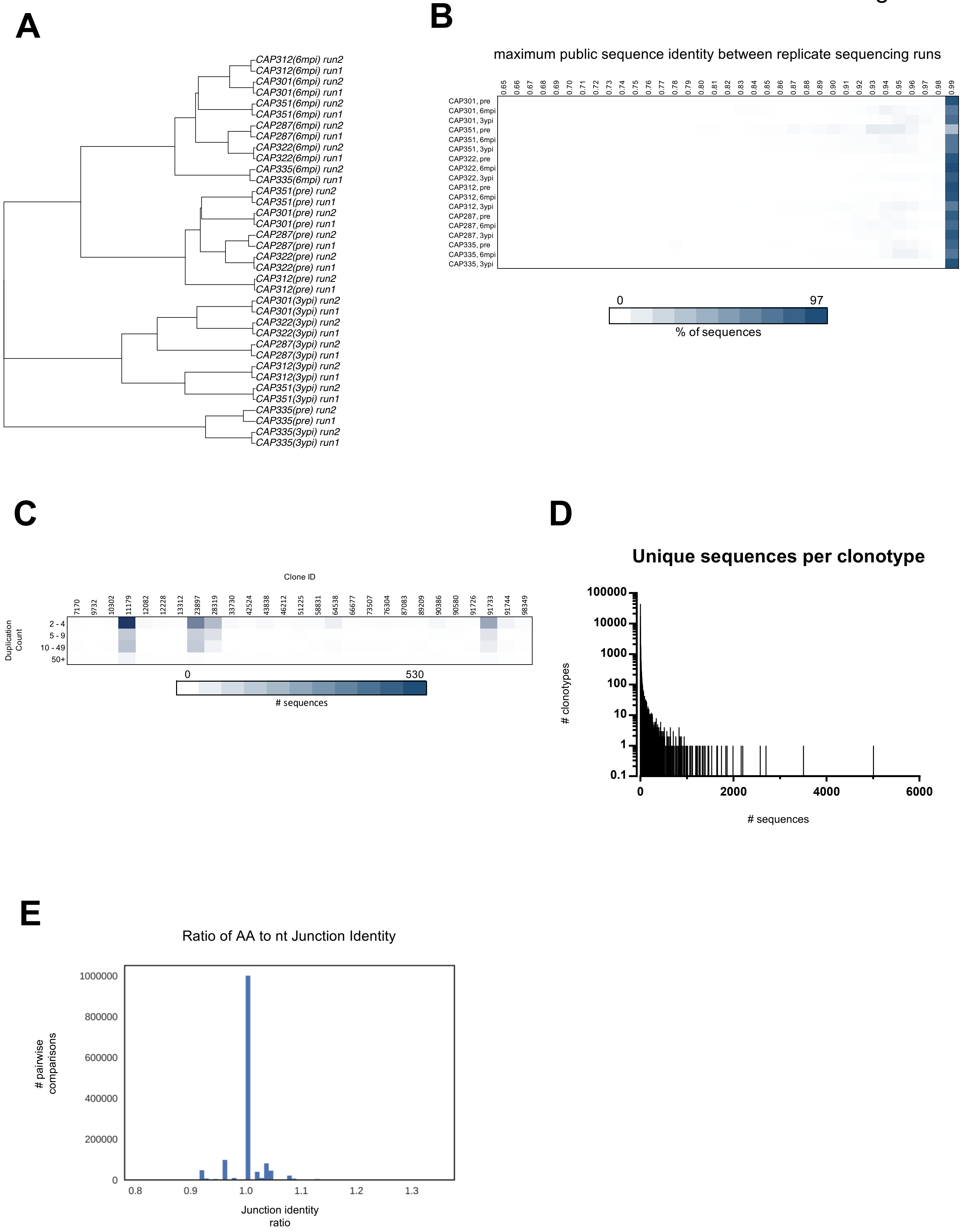
A
CAP312(6mpi) run2
CAP312(6mpi) run1
CAP301(6mpi) run2
CAP301(6mpi) run1
CAP351(6mpi) run2
CAP351(6mpi) run1
CAP287(6mpi) run2
CAP287(6mpi) run1
CAP322(6mpi) run2
CAP322(6mpi) run1
CAP335(6mpi) run2
CAP335(6mpi) run1
CAP351(pre) run2
CAP351(pre) run1
CAP301(pre) run2
CAP301(pre) run1
CAP287(pre) run2
CAP287(pre) run1
CAP322(pre) run2
CAP322(pre) run1
CAP312(pre) run2
CAP312(pre) run1
CAP301(3ypi) run2
CAP301(3ypi) run1
CAP322(3ypi) run2
CAP322(3ypi) run1
CAP287(3ypi) run2
CAP287(3ypi) run1
CAP312(3ypi) run2
CAP312(3ypi) run1
CAP351(3ypi) run2
CAP351(3ypi) run1
CAP335(pre) run2
CAP335(pre) run1
CAP335(3ypi) run2
CAP335(3ypi) run1
B
maximum public sequence identity between replicate sequencing runs
CAP301, pre
CAP301, 6mpi
CAP301, 3ypi
CAP351, pre
CAP351, 6mpi
CAP351, 3ypi
CAP322, pre
CAP322, 6mpi
CAP322, 3ypi
CAP312, pre
CAP312, 6mpi
CAP312, 3ypi
CAP287, pre
CAP287, 6mpi
CAP287, 3ypi
CAP335, pre
CAP335, 6mpi
CAP335, 3ypi
0
97
% of sequences
C
Clone ID
Duplication Count
2 - 4
5 - 9
10 - 49
50+
0
530
sequences
D
Unique sequences per clonotype
clonotypes
sequences
E
Ratio of AA to nt Junction Identity
pairwise comparisons
Junction identity ratio

Figure S4, Related to STAR Methods. Analysis of sequencing data properties.

a. Repertoire similarity of technical replicates for the same donor was very high, as assessed using the repertoire similarity metric reported by VDJtools as described in (Shugay et al., 2015). An all-vs-all comparison of repertoires was performed, and hierarchical clustering was performed based on repertoire similarity as measured by overlap of identical CDRH3 sequences. In all cases, replicates were most similar to each other.
b. For each public sequence (CDR1 through CDR3) in each sample (rows) of the original sequencing run, the maximum identity (columns) found in the corresponding sample of the duplicate sequencing run was calculated. Heatmap color intensity is proportional to the density of antibody clonotypes for each binned identity value for each sample. Bin labels indicate the left bound, with all bins right-open except 0.99, which is inclusive of 1.00.
c. Duplication count (rows) per VDJ sequence in each of the 27 most public clonotypes (columns).
d. Number of clonotypes (y-axis) with given number of member unique VDJ sequences (x-axis), for the 70% junction identity threshold plus matched V- and J- gene, using complete linkage clustering.
e. Number of pairwise comparisons of sequences (y-axis) with given ratios of amino acid junction identity and nucleotide junction identity (x-axis) between pairs of members of the 27 most public clonotypes. Only member sequences belonging to the same clonotype were compared to one another.

Donor ID	Date of Infection	Visit Classification	Visit weeks post infection (wpi)	Visit Date	Medical Events	Viral Load	Neutralization Breadth (%)
CAP301	31-May-08	Pre-infection	-9	31-Mar-08	(1) Hep B core IgG positive at enrolment on 25-Jun-08. (2) 5 episodes of documented STIs. (3) Other minor ailments reported: tonsillitis, axillary abscess, scleral lesions.	—	
		6mpi	28	11-Dec-08		6210	
		3ypi	171	13-Sep-11		< 20	11
CAP351	14-May-09	Pre-infection	-18	8-Jan-09	(1) PTB in 2007-2008. Completed treatment in Aug 2008. (2) TB episode prior to HIV acquisition. (3) Herpes zoster 24-Mar-11. (4) Recurrent upper respiratory tract infections.	—	
		6mpi	22	15-Oct-09		33600	
		3ypi	144	13-Feb-12		38026	6
CAP335	24-Dec-08	Pre-infection	-30	26-May-08	Generally well throughout	—	
		6mpi	23	3-Jun-09		11500	
		3ypi	172	10-Apr-12		16955	0
CAP287	1-Dec-07	Pre-infection	-2	15-Nov-07	(1) Generally well. (2) Trichomas vaginalis July 2008. (3) Chronic hypertension. (4) Mild anaemia with B12 defiiency in pregnancy. (5) Pregnancy Jan to Oct 2009.	—	
		6mpi	28	12-Jun-08		39500	
		3ypi	162	11-Jan-11		4140	61
CAP312	29-Jul-08	Pre-infection	-26	29-Jan-08	(1) PV discharge and Lymphadenopathy noted at numerous Phase 2-4 physical exam CRFs (2008-2011). (2) Bartholin's abcess. Admitted to hospital for marsupialisation of abscess. (3) T. vaginalis 05-Aug-10. (4) Miscarriage 15-Jul-11. History of passing clots and pregnancy test positive, outcome unknown. (5) Recurrent diarrhoea in 2014.	—	
		6mpi	25	20-Jun-09		10400	
		3ypi	157	2-Aug-11		546	61
CAP322	1-Jul-08	(Pre-infection)	2	15-Jul-08	(1) Generally well. (2) HIV related cervical lymphadenopathy Sept 2010 that resolved spontaneously. (3) Hep B cAb positive, Hep B sAg negative May 2010. (4) Hep B cAb negative Apr 2011. (5) Normal liver function tests throughout.	—	
		6mpi	27	7-Jan-09		34300	
		3ypi	161	4-Aug-11		5386	39
CAP248	23-Mar-05	5.9ypi	298	8-Feb-11	Generally well throughout	26509	59 (at 3ypi)
CAP314	9-Jun-08	2ypi	115	24-Aug-10	(1) History of TB. (2) Pneumonia in 2010. (3) Herpes Zoster in 2010. (4) History of Candidiasis.	25805	44

Table S1, Related to STAR Methods. Clinical features and timepoints of sample collection for the donors in this study.

serum	VRC01-like	b12-like	HJ16-like	8ANC195-like	PG9-like	PGT128-like	2F5-like	10E8-like	35O22-like	PGT151-like	Median of delineation scores
CAP287		0.17	0.18			0.64					0.0000
CAP312	0.03	0.33	0.21		0.28					0.14	0.0175
CAP322	0.24	0.22				0.23		0.09		0.21	0.0485

Table S2, Related to STAR Methods. Neutralization fingerprinting for CAP287, CAP312, and CAP322 indicating differing antibody specificities among subjects with broadly neutralizing serum.

PID	Timepoint	Sample #	Sum of Filtered Read Count	# Unique VDJ Sequences	# Clonotypes (70%)	# Public Clonotypes	# Private Clonotypes	# Unique VDJ Sequences in Public Clonotypes
CAP301	pre-infection	12	1435042	85957	5017	356	4661	20252
	6mpi	11	1430714	83750	4487	380	4107	2539
	3ypi	7	784491	48700	6420	610	5810	4526
CAP351	pre-infection	16	1180638	65297	4712	253	4459	695
	6mpi	8	1709720	101426	6204	329	5875	2774
	3ypi	6	1085531	70554	13832	916	12916	3277
CAP335	pre-infection	14	525443	28110	3527	252	3275	5538
	6mpi	5	802391	50823	7349	524	6825	4206
	3ypi	1	1095128	68986	6609	470	6139	22722
CAP287	pre-infection	10	1536756	53242	1872	129	1743	12324
	6mpi	15	1105900	57990	5150	518	4632	6181
	3ypi	2	1178014	58283	2214	147	2067	6116
CAP312	pre-infection	9	1265235	79683	3839	297	3542	32392
	6mpi	13	1134878	54980	2369	142	2227	10426
	3ypi	3	1808428	106429	8438	420	8018	3122
CAP322	(pre-infection)	17	1523989	96935	9245	602	8643	6588
	6mpi	18	1155960	60456	3004	202	2802	21487
	3ypi	4	1464792	94790	14920	1005	13915	8501
**Total**			**22223050**	**1266391**	**103475**	**3515**	**99960**	**172755**

Table S3, Related to STAR Methods. Sequencing depth information and counts of public and private clonotypes for each sample, for the 70% junction region identity threshold.

A

CAP256-VRC26	VRC26.12	VRC26.11	VRC26.10	VRC26.09	VRC26.08	VRC26.07	VRC26.06	VRC26.05	VRC26.04	VRC26.03	VRC26.02	VRC26.01
VRC26.12		0.615	0.615	0.609	0.634	0.769	0.725	0.692	0.743	0.743	0.615	0.384
VRC26.11	0.615		0.743	0.609	0.585	0.641	0.575	0.692	0.666	0.666	0.666	0.333
VRC26.10	0.615	0.743		0.658	0.609	0.666	0.625	0.769	0.692	0.692	0.897	0.435
VRC26.09	0.609	0.609	0.658		0.926	0.78	0.547	0.658	0.804	0.804	0.634	0.341
VRC26.08	0.634	0.585	0.609	0.926		0.731	0.571	0.682	0.756	0.756	0.585	0.317
VRC26.07	0.769	0.641	0.666	0.78	0.731		0.65	0.641	0.948	0.923	0.692	0.384
VRC26.06	0.725	0.575	0.625	0.547	0.571	0.65		0.65	0.65	0.65	0.65	0.4
VRC26.05	0.692	0.692	0.769	0.658	0.682	0.641	0.65		0.666	0.666	0.743	0.487
VRC26.04	0.743	0.666	0.692	0.804	0.756	0.948	0.65	0.666		0.974	0.692	0.384
VRC26.03	0.743	0.666	0.692	0.804	0.756	0.923	0.65	0.666	0.974		0.692	0.384
VRC26.02	0.615	0.666	0.897	0.634	0.585	0.692	0.65	0.743	0.692	0.692		0.435
VRC26.01	0.384	0.333	0.435	0.341	0.317	0.384	0.4	0.487	0.384	0.384	0.435	

B

CH235	CH235.10	CH235.11	CH235.12	CH235.13	CH235.6	CH235.7	CH235.8	CH235.9
CH235.10		0.588	0.705	0.705	0.47	0.647	0.529	0.705
CH235.11	0.588		0.588	0.588	0.352	0.529	0.411	0.588
CH235.12	0.705	0.588		1	0.529	0.882	0.647	1
CH235.13	0.705	0.588	1		0.529	0.882	0.647	1
CH235.6	0.47	0.352	0.529	0.529		0.588	0.529	0.529
CH235.7	0.647	0.529	0.882	0.882	0.588		0.647	0.882
CH235.8	0.529	0.411	0.647	0.647	0.529	0.647		0.647
CH235.9	0.705	0.588	1	1	0.529	0.882	0.647	

Table S4, Related to STAR Methods. Intra-clonal junction region identity of known broadly neutralizing HIV antibodies.

a. Pairwise junction identity matrix of members of the VRC26 broadly neutralizing antibody lineage (Doria-Rose et al., 2014b).

b. Pairwise junction identity matrix of members of the CH235 broadly neutralizing antibody lineage (Bonsignori et al., 2016).

References

Cheadle, C., Vawter, M.P., Freed, W.J., and Becker, K.G. (2003). Analysis of microarray data using Z score transformation. J Mol Diagn. 5, 73-81.

Crooks, G.E., Hon, G., Chandonia, J.M., and Brenner, S.E. (2004). WebLogo: a sequence logo generator. Genome Res. 14(6), 1188-90.

Shugay, M., Bagaev, D.V., Turchaninova, M.A., Bolotin, D.A., Britanova, O.V., Putintseva, E.V., Pogorelyy, M.V., Nazarov, V.I., Zvyagin, I.V., Kirgizova, V.I., et al. (2015). VDJtools: Unifying Post-analysis of T Cell Receptor Repertoires. PLoS Comput Biol. 11, e1004503.

Doria-Rose, N.A., Bhiman, J.N., Roark, R.S., Schramm, C.A., Gorman, J., Chuang, G.Y., Pancera, M., Cale, E.M., Ernandes, M.J., Louder, M.K., et al. (2015). New Member of the V1V2-Directed CAP256-VRC26 Lineage That Shows Increased Breadth and Exceptional Potency. J Virol. 90, 76-91.

Bonsignori, M., Zhou, T., Sheng, Z., Chen, L., Gao, F., Joyce, M.G., Ozorowski, G., Chuang, G.Y., Schramm, C.A., Wiehe, K., et al. (2016). Maturation Pathway from Germline to Broad HIV-1 Neutralizer of a CD4-Mimic Antibody. Cell. 165, 449-63.
